# Tuberculosis

**Published:** 1894-05-19

**Authors:** Wm. Calwell

**Affiliations:** Physician to the Ulster Hospital for Women and Children, &c.


### Medical Progress and Hospital Clinics.

[*The Editor will be glad to receive offers of co-operation and contributions from members of the profession. All letters should be addressed* o The Editor, The Lodge, Porchester Square, London, W.]

### TUBERCULOSIS.

Wm. Calwell, M.D., Physician to the Ulster Hospital for Women and Children, &c.

The question of tubercle is one of such momentous importance that it can never become uninteresting, nor the study of it unprofitable. Nothing startlingly new has been brought to light lately, still it is well perhaps every now and then to reconsider our position with regard to it, and to summarise our knowledge so that we may see more clearly what has been done and what is to be done. Let us then briefly state what we actually know, and putting away all preconceived notions, infer what we should do.

We must grant the existence of the bacillus tuberculosis, which exhibits a definite form and definite reactions to certain staining agents. It is found wherever tubercular degeneration in its early stages is found, and it is not found in animal tissue without tubercular degeneration being set up sooner or later. This pathological process, *i.e.*, tubercular degeneration, is always one and the same, no matter in what animal nor in what tissue it is present. It varies, but the variations are those of degree according to the tissue, and probably according to the vitality of the microbe itself, and not of kind. The disease is found in greatest abundance in man, next in dairy cows, but also in nearly every domestic animal, such as pigs, hens, &c. Some, however, such as goats, enjoy a well-marked immunity. Wild animals (and, indeed, savage man) are free, but speedily succumb on being brought into captivity; and herbivora suffer more than carnivora. The bacillus can be cultivated in special artificial culture when kept at the temperature of the body, and can be studied through successive generations; the cultures show a peculiar and characteristic growth, and the organism does not seem to lose its virulence. We know also that it exists potentially in effete tubercular degenerations, probably as spores, which are not revealed to the microscope, because matter from such a condition when inoculated into a culture begets the ordinary growth; in its early stages such matter does present the microbe. The natural history of the organism is interesting. It is one of the Fission fungi, and is very hardy and tenacious of life; it can exist for a month in dried dust; it can be frozen and come to life again, and be raised to temperature far beyond that borne by man and still survive; it resists the action of most of the ordinary antiseptics in the strength in which they are ordinarily employed; and when a lotion of required strength is used, a much longer immersion is necessary that is allowed in practice.

The microbe does not multiply outside the body, unless in specially prepared media, kept at the proper temperature. Existence in the dust of our houses or roads is most probable, nearly certain; proliferation under those conditions does not occur. Again, with the exception of one or two pathological curiosities, the newly born are free from this disease, the inference from which is that admission by the microbe is gained after birth.

If these statements are true, and they seem to be generally acknowledged by those most competent to judge, we are driven to the conclusion that a patient suffering from tubercle must have received the initial poison directly or indirectly from some man or animal similarly affected.

There are three ways by which the bacillus may gain entrance: 1, inhalation; 2, ingestion; 3, inoculation. The last is rare except for experimental purposes, and may be dismissed on being mentioned. The first is suspected to be by far the commonest. If we dry tubercular matter and cause an animal to inspire the dust so formed, the experiment results in a surprisingly large number of cases in the development of consumption; the details of the procedure may be varied, but a fatal termination is of sufficient frequency to warrant our conclusion that the inspired dust is the cause of the consumption. A large amount of evidence has been adduced that the analogy does not hold with man; nurses in consumption hospitals mingle among consumptive patients, and yet do not develop consumption, but then in well regulated institutions of this kind patients are not allowed to expectorate freely on the floors, and it is very improbable that a nurse from a consumptive family would undertake the duties of that station. Statistics given by Cornet in cases where these special precautions were not taken tend strongly to support the analogy, and we must acknowledge it to be possible and very probable. Anyone who has examined the sputa of a consumptive patient must realise at once the dreadful infectivity of this excretion. Animals fed on tubercular matter, that is with portions of diseased tissue mixed with their food, likewise develop some abdominal tubercular affection sooner or later. This is especially the case with the very young fed with infected milk. A litter of young pigs nourished on the milk of a cow whose udder is diseased die off, and die from the same disease, namely, that from which the cow is suffering. Does the analogy hold with mankind? The facts are more than suspicious. Tubercular disease in children is most frequent during the second, third, and fourth year of life, and this is the exact time that children drink uncooked cow's milk. Butchers are a very estimable set of men, but it is well known they put everything into sausages, and more than once bits of diseased glands, &c., have been found in these dainties. It is done from ignorance, not now, perhaps, so much as formerly. We cannot prove that such a sausage, insufficiently cooked, will cause a tubercular growth in the abdomen, but we must acknowledge its possibility—even its strong probability—if repeated in numerous instances.

These, then, according to modern light, seem to be the modes of infection of human beings or of animals. Air contaminated by the dried tuberculous excreta of a diseased man or animal is breathed by man or animal; food, similarly contaminated, is eaten by man or animal, and the result is infection, in what proportion of cases we cannot yet say. Once the microbe has established a stronghold in living tissue, it multiplies. If the patient is healthy, this growth may be checked and the lesion dries up; if the patient is below par, or of a tubercular stock, it spreads, colonies are developed in the surrounding tissue by means of the lymphatic, or in distant parts of the body by means of the blood circulation; a consumptive patient may inoculate one lung from the other, or may set up abdominal trouble by swallowing the sputa.

The other great factor to be considered in this subject is predisposition, especially if it is hereditary; this predisposition may show itself in an outward and visible manifestation, as the strumous or scrofulous type, or it may not. Besides heredity we have exhaustion from over lactation, bad ventilation, certain confining occupations and trades, damp low-lying districts, overcrowding; but we shall not get consumption all the same unless the bacillus is present.

Our object then is twofold, whether we deal with individuals or with nations; remove the predisposing causes and destroy the sources of infection. The laws of hygiene, physiological righteousness, cover the former; the latter is becoming more practical every year as the truth spreads. The excreta of every tubercular organ is deadly, especially the sputa of consumptive patients. The milk and flesh of tubercular cows and of other domestic animals is dangerous. Once these two facts are known the course to be pursued will soon be clear.

